# On the Effect of pH, Temperature, and Surfactant Structure on Bovine Serum Albumin–Cationic/Anionic/Nonionic Surfactants Interactions in Cacodylate Buffer–Fluorescence Quenching Studies Supported by UV Spectrophotometry and CD Spectroscopy

**DOI:** 10.3390/ijms23010041

**Published:** 2021-12-21

**Authors:** Krzysztof Żamojć, Dariusz Wyrzykowski, Lech Chmurzyński

**Affiliations:** Faculty of Chemistry, University of Gdansk, Wita Stwosza 63, 80-308 Gdansk, Poland; dariusz.wyrzykowski@ug.edu.pl (D.W.); lech.chmurzynski@ug.edu.pl (L.C.)

**Keywords:** bovine serum albumin, surfactants, binding properties, steady-state fluorescence spectroscopy, fluorescence quenching

## Abstract

Due to the fact that surfactant molecules are known to alter the structure (and consequently the function) of a protein, protein–surfactant interactions are very important in the biological, pharmaceutical, and cosmetic industries. Although there are numerous studies on the interactions of albumins with surfactants, the investigations are often performed at fixed environmental conditions and limited to separate surface-active agents and consequently do not present an appropriate comparison between their different types and structures. In the present paper, the interactions between selected cationic, anionic, and nonionic surfactants, namely hexadecylpyridinium chloride (CPC), hexadecyltrimethylammonium bromide (CTAB), sodium dodecyl sulfate (SDS), polyethylene glycol sorbitan monolaurate, monopalmitate, and monooleate (TWEEN 20, TWEEN 40, and TWEEN 80, respectively) with bovine serum albumin (BSA) were studied qualitatively and quantitatively in an aqueous solution (10 mM cacodylate buffer; pH 5.0 and 7.0) by steady-state fluorescence spectroscopy supported by UV spectrophotometry and CD spectroscopy. Since in the case of all studied systems, the fluorescence intensity of BSA decreased regularly and significantly under the action of the surfactants added, the fluorescence quenching mechanism was analyzed thoroughly with the use of the Stern–Volmer equation (and its modification) and attributed to the formation of BSA–surfactant complexes. The binding efficiency and mode of interactions were evaluated among others by the determination, comparison, and discussion of the values of binding (association) constants of the newly formed complexes and the corresponding thermodynamic parameters (Δ*G*, Δ*H*, Δ*S*). Furthermore, the influence of the structure of the chosen surfactants (charge of hydrophilic head and length of hydrophobic chain) as well as different environmental conditions (pH, temperature) on the binding mode and the strength of the interaction has been investigated and elucidated.

## 1. Introduction

Due to a huge variety of industrial, biological, medical, and technical applications of surfactants in the fields of chemicals, detergents, cosmetics, foods, and pharmaceuticals [1,2,3,4,5,6,7,8,9,10], their interactions with proteins have been in recent years extensively studied, mainly in terms of factors that determine the association extent as well as modifications of the protein physicochemical and functional properties (in the case of enzymes, transport proteins, and toxins) caused by association-elicited conformational changes [11,12,13,14,15,16,17].

The macromolecule–surfactant interactions depend on many factors, among which the type of surfactant species (monomer, micelles) presented in the system under study [18,19], the chemical structure of a surfactant (anionic, cationic, amphoteric, or nonionic) [20], its architecture [21,22], as well as experimental conditions (pH, temperature) [23,24] play crucial roles.

**Figure 1:**
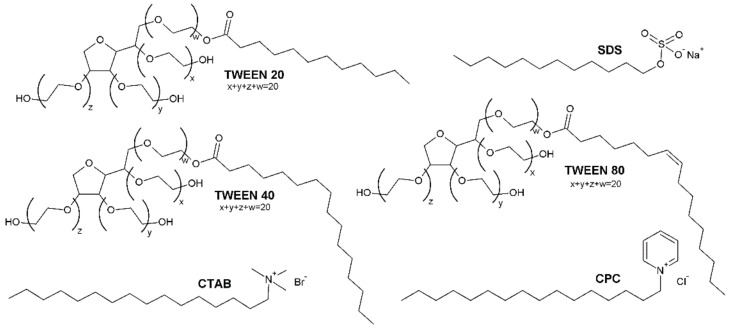
Molecular structures of the studied surfactants.

In this paper, we report the interactions between selected surfactants (the structures are presented in Figure 1) with bovine serum albumin studied under various experimental conditions. Contrary to many previous reports—as most of the studies concern protein denaturation at surfactants concentrations much above their critical micelle concentration [25,26,27,28,29]—we have focused on low concentrations of detergents. Furthermore, we have used simultaneously surfactants belonging to three main groups (cationic, anionic, and nonionic), although most of the comprehensive studies refer to ionic ones [30,31,32,33,34]; the interactions with nonionic surfactants, used normally in formulations due to stabilizing properties [35], are found to be weaker, and the interpretation of results is somehow more difficult [31,36]. Finally, to the best of our knowledge, this is the first report on the molecular interactions between a group of conventional surfactants and BSA elucidated in cacodylate (CACO) buffer. Its relatively wide useful buffering range enables performing the measurements in the same buffer solution and at different pH (5.0 and 7.0).

In our studies, we have used steady-state fluorescence spectroscopy (supported by UV spectrophotometry and CD spectroscopy), which is regarded as an important tool for the studies of various chemical individuals (among others metal complexes [37], inclusion complexes [38], gases [39], thiols [40], radicals [41], amino acids [42], nanoparticles [43]) as well as an effective technique to establish conformational modifications and interactions of different biomolecules in a solution [44].

The presented results have important implications to understand the influence of commonly used surfactants on the functionality of proteins in modern branches of chemistry. The investigations of structural features of the surfactants that govern their affinity toward a protein may be helpful for the development of new drug delivery systems for improved medical treatments [45,46,47]. Consequently, it was the main reason that prompted us to embark on these studies.

## 2. Results and Discussion

The bovine serum albumin (BSA) in cacodylate buffer (CACO) exhibits an emission peak with a maximum at 348 nm (due to tryptophan residues). On the addition of all studied surfactants, the peak becomes more or less blue shifted (up to 332 nm) with a clear decrease in the quantum yield (Figure S1 in Electronic Supplementary Information). The observed shift and alteration in the fluorescence intensity of the attained band provide essential information about events that affect the microenvironment surrounding the aromatic residues in the protein molecule (such as protein conformational transitions, ligand binding, denaturation, etc.) [48] and may be a result of conformational changes in BSA structure, which lead to the exposure of intrinsic fluorophores to more hydrophobic environment (the change in the environment of tryptophan and an increase in hydrophobicity in the vicinity of this residue due to the presence of the alkyl chains of the surfactant molecules) [49]. On the other hand, at pH 7.4, anionic D and E residues remain near W^134^, which lies at the surface of BSA (subdomain IB) [50]. Consequently, the possible binding of the studied surfactants by electrostatic interactions under experimental conditions (namely at pH 7.0) cannot be ruled out. Figure S2 in the Electronic Supplementary Information presents the UV absorption spectra and difference UV absorption spectra of BSA (2 µM) in the presence of increasing concentrations of the studied surfactants in 10 mM CACO buffer of pH 5.0 and 7.0 at 298 K. From the inspection of these spectra, it can be clearly observed that—in the studied range of concentrations—all examined surfactants exhibit a slight but noticeable influence on the UV spectrum of the free albumin. When the concentration of the surfactant increases, the band with a maximum at approximately 220 nm gradually appears with no or a negligible impact on the band attributed to the tryptophan residue (namely 275 nm). The findings from the analysis of both UV absorption and fluorescence emission spectra may be indicative of the formation of BSA–surfactants complexes.

**Figure 2:**
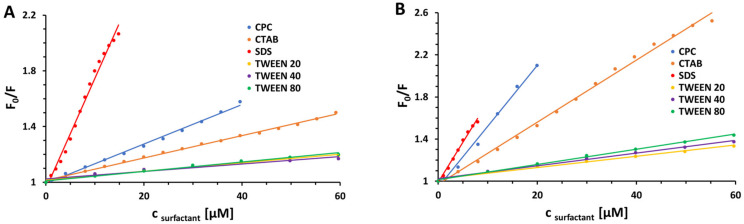
Stern–Volmer plots for the steady-state fluorescence quenching of BSA by different surfactants in 10 mM CACO buffer of pH 5.0 (**A**) and 7.0 (**B**) at 298 K.

**Figure 3:**
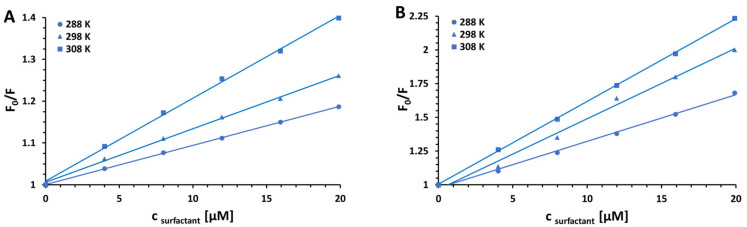
Stern–Volmer plots for the steady-state fluorescence quenching of BSA by CPC in 10 mM CACO buffer of pH 5.0 (**A**) and 7.0 (**B**) at various temperatures (288 K, 298 K, and 308 K).

To get an insight into qualitative and quantitative assessment of the interactions between the BSA and the surfactants, the obtained results were analyzed according to the Stern–Volmer equation:

(1) $$\frac{F_{0}}{F}=1+K_{\mathrm{SV}}\left[Q\right]=1+k_{q}\tau_{0}\left[Q\right]$$

where F_0_ and F denote the steady-state fluorescence intensities in the absence and presence of a quencher (surfactant), [Q] is the quencher concentration, *K*_SV_ is the Stern–Volmer quenching constant, *k*_q_ is the bimolecular quenching rate constant, while τ_0_ is the average lifetime of the fluorophore (BSA) in the absence of quencher [51,52,53,54]. The graphs of $\frac{F_{0}}{F}$ versus [Q] plotted for the steady-state fluorescence quenching of BSA tryptophan residues by various surfactants under experimental conditions according to the Stern–Volmer equation are shown in Figure 2 and Figure 3. Consequently, Table 1 presents the newly determined values of Stern–Volmer quenching constants (*K*_SV_) along with linear correlation coefficients (R^2^) for Stern–Volmer plots and bimolecular quenching rate constants (*k*_q_) recovered for the studied systems. The latter ones were calculated based on the values of the average fluorescence lifetimes τ_0_ of free BSA equal to approximately 6 ns at pH 7.0 (phosphate buffer) and 5.7 ns at pH 5.0 (acetate buffer) [55].

In the studied concentration range, strictly straight lines were obtained with no deviations from the observed linearity. To get a deeper insight into the mechanism of fluorescence quenching, the newly determined bimolecular quenching rate constants were compared with the maximum scattering collision quenching rate constant, which for the interactions of various quenchers with biopolymer in aqueous media is equal to 2.0 × 10^10^ M^−1^ s^−1^ [56,57,58]. The estimated values of *k*_q_ for all surfactants are of the order 10^11^–10^13^ M^−1^ s^−1^, which is approximately 23–700-fold higher than the mentioned maximum value possible for diffusion controlled quenching rate constant. Since the values of bimolecular quenching rate constants are considered to be definitive in differentiating between dynamic and static quenching mechanisms [59,60], the predominant role of ground-state complexation (static quenching) in the investigated systems was affirmed. The observed changes in fluorescence emission of BSA induced by low surfactant concentrations are probably due to the changes of W^131^ residue orientation and contacts with neighboring residues—some residues in close contact with the tryptophan indole group are known to effectively quench the fluorescence by a static quenching mechanism [61,62,63]. Interestingly, the results presented in Figure 2 revealed that the quenching constants and thus the stabilities of the resulting BSA–surfactant complexes increase in proportion to the increase in temperature [64,65,66]. This phenomenon suggests an endothermic nature of the investigated interactions. The above assumption has been subsequently verified by examining the thermodynamic parameter of the reactions under study.

For a static quenching interaction, when small molecules bind independently to a set of equivalent sites of a protein, the equilibrium between free and bound molecules is given by the following modified version of the Stern–Volmer equation [67,68]:

(2) $$\log\left[\frac{F_{0}-F}{F}\right]=\log K_{a}+n\log\left[Q\right].$$

**Figure 4:**
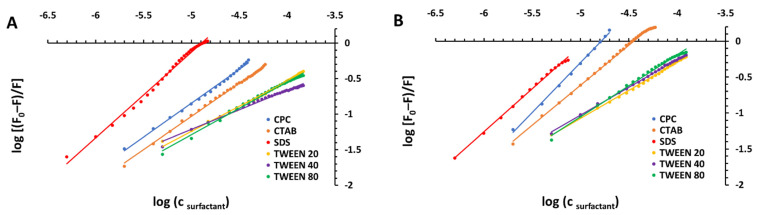
Modified Stern–Volmer plots for the steady-state fluorescence quenching of BSA by different surfactants in 10 mM CACO buffer of pH 5.0 (**A**) and 7.0 (**B**) at 298 K.

**Figure 5:**
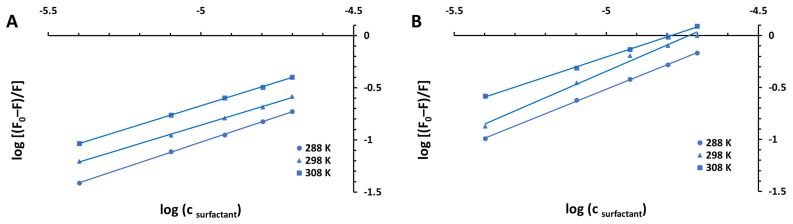
Modified Stern–Volmer plots for the steady-state fluorescence quenching of BSA by CPC in 10 mM CACO buffer of pH 5.0 (**A**) and 7.0 (**B**) at various temperatures (288 K, 298 K, and 308 K).

Thus, the apparent binding constant (*K*_a_) and the number of binding sites (*n*) can be calculated from the intercepts and the slopes of the linear plots of $\log\left[\frac{F_{0}-F}{F}\right]$ versus log[Q], respectively (Figure 4 and Figure 5). These values have been obtained (as the averages of three independent experiments) and are shown in Table 2, along with linear correlation coefficients (R^2^) recovered for the studied systems.

The value of *K*_a_ is particularly relevant for the understanding of the distribution of the drug in plasma, since weak binding can lead to a short lifetime or a poor distribution, while strong binding is responsible for the reduction in the plasmatic distribution of free drug [69,70]. The *K*_a_ values obtained for the studied BSA complexes suggest relatively high/medium binding affinity to the protein for ionic surfactants (mainly at higher pH) and significantly lower binding affinity for nonionic ones when compared to other known strong biomolecule–ligand complexes with binding constants ranging from 10^5^ to 10^8^ M^−1^ [71,72,73]. However, lower binding constants (10^2^−10^4^ M^−1^), which indicate a very weak interaction between the ligand and the protein, have been reported for several other protein–ligand complexes as well [74,75,76]. Since the interactions between studied ionic surfactants and BSA are quite significant (mainly at pH 7.0) and the effect of temperature is rather small, it shows that these compounds can be stored and transported by the protein in the body [77]. For most of the surfactant–serum albumin complexes, the value of *n* was approximately equal to one, indicating the existence of just a single binding site in BSA for the studied compounds [78,79].

Generally, there have been suggested two main types of interactions between surfactants and proteins, namely specific binding by electrostatic attraction/repulsion between oppositely/equally charged surfactant head groups and sites of the proteins or the cooperative association of alkyl chains to the protein via hydrophobic affinity [80]. It can be observed that the fluorescence quenching of BSA is clearly pH dependent; decreasing pH results in a noticeable reduction in the quenching efficiency (Figure 2, Table 1) and, consequently, binding affinity (Figure 4, Table 2). It proves that the protonation of the protein residues affects the binding of the surfactants (except using SDS, where the change in pH does not affect its affinity toward the first site of the protein). In the case of—for example—cationic surfactants, an increase in pH from 5.0 to 7.0 probably favors interactions between negatively charged groups in the protein and CPC/CTAB. At pH 5.0, the protein is packed in a more compact form (pH 5.0 is close to the isoelectric point; pI = 5.2 [81]), which reduces the accessibility of the surfactants to the hydrophobic cavities in the binding sites. Conversely, increasing the pH makes the protein more flexible and open in its structure [82]—more mobile peptide chains contribute to an increase in the accessibility of the surfactants to the hydrophobic core. On the other hand, higher values of Stern–Volmer quenching constants were obtained for SDS in comparison with the ones for CPC and CTAB (particularly at pH 7.0; above the isoelectric point). It is in a good agreement with the literature, and the possible reason may lie in the surfactant features. Anionic surfactants interact strongly with proteins and cause their denaturation, which is possible due to the unfolding of proteins induced by surfactants. Comparing with anionic surfactants, cationic ones exhibit a lower tendency to interact with proteins mainly as a consequence of a smaller relevance of electrostatic interaction at the pHs of interest [77,83,84]. The observed phenomenon can also suggest that the charges of the surfactant head group modulate the interactions only partially, and the hydrophobic interactions of surfactant methylene chains are responsible for binding as well.

Apart from pH, the impact of the structure of the surfactant on the quenching intensity was also considered. The comparison of bimolecular quenching rate constants obtained for BSA–CTAB and BSA–CPC systems led to an evaluation of the influence of the pyridinium moiety, which was found to be an efficient fluorescence quencher that could reduce the intrinsic protein fluorescence (which is in good agreement with the literature [85]). Furthermore, the results obtained for TWEEN 20, TWEEN 40, and TWEEN 80 revealed no significant changes in the strength of interactions between these surfactants and BSA, which proves that the length of the alkyl chain can be of the slightest importance. On the other hand, at pH 7.0, the following dependence can be observed: *K*_a_ TWEEN 20 < *K*_a_ TWEEN 40 < *K*_a_ TWEEN 80. This trend is expected as the stability of the resulting complexes increases with the lengthening of the hydrophobic alkyl chain of the surfactant [45,50]. Thus, the formation of the BSA complexes with nonionic surfactants seems to be an entropy-driven process.

**Figure 6:**
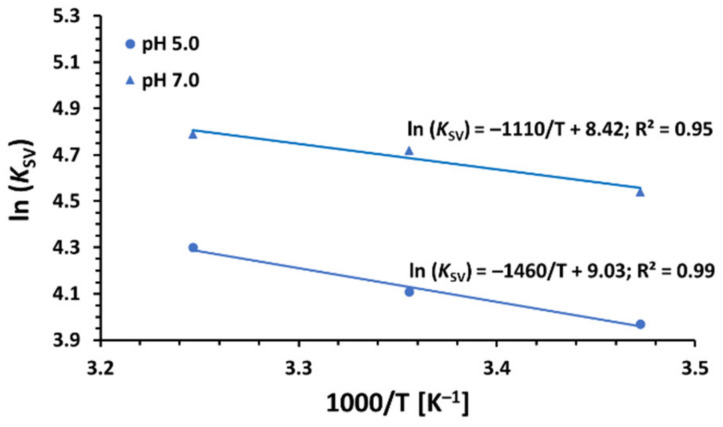
Van’t Hoff plots for the steady-state fluorescence quenching of BSA by CPC in 10 mM CACO buffer of pH 5.0 and 7.0.

The acting forces for binding between a small molecule and protein may include hydrophobic and electrostatic interactions, van der Waals interactions, hydrogen bonds, etc. [86,87]. The noncovalent interaction forces between various ligands and biomolecules can be described by thermodynamic parameters, which can be calculated from the van’t Hoff equation:

(3) $$\ln K_{\mathrm{SV}}=-\frac{\Delta H}{\mathrm{RT}}+\frac{\Delta S}{R}$$

where Δ*H* and Δ*S* are enthalpy change and entropy change, respectively, R is the gas constant, and T denotes the absolute temperature [88]. The plot of ln*K*_SV_ (for CPC) measured at three different temperatures vs. $\frac{1}{T}$ is linear within the experimental error (Figure 6), which allowed one to calculate Δ*H* and Δ*S* for the quenching process.

It has been reported that the positive Δ*H* and Δ*S* values are associated with hydrophobic interactions playing a major role in the binding reaction; negative Δ*H* and Δ*S* values are correlated with van der Walls interactions and hydrogen bonding, while electrostatic forces usually make Δ*H* ≈ 0 or Δ*H* < 0 and Δ*S* > 0 [89]. The negative sign for free energy change of quenching ΔG ($\Delta G=\Delta H-T\Delta S=-\mathrm{RTln}K_{\mathrm{SV}}$) for all studied surfactants indicates the spontaneity of their binding with bovine serum albumin in the concentration range employed [49]. The positive Δ*H* (12.1 kJ mol^−1^ for pH 5.0 and 9.23 kJ mol^−1^ for pH 7.0) and Δ*S* (75.1 J mol^−1^ K^−1^ for pH 5.0 and 70.0 J mol^−1^ K^−1^ for pH 7.0) indicate that the interaction between BSA and CPC is mainly entropy-driven, the enthalpy is unfavorable for it, and the hydrophobic forces play a major role with less dominating hydrogen bonds formation. The calculated positive values of the enthalpy changes stay in line with the observed increase in the *K*sv values with the increase in temperature, confirming a static quenching mechanism of the protein–surfactant complex formation [90,91].

The far-UV CD spectroscopy of BSA and the resulting BSA–surfactant complexes were employed for monitoring the changes in the secondary structure of the investigated albumin. For all systems studied, two negative bands characteristic for the α-helix structure are present in the CD spectra at 208 nm (the *p* → p* transition) and 222 nm (the *n* → p* transition) (Figure S3 in Electronic Supplementary Information) [92]. The conformational contributions of α-helix, β-sheet, turns, and unordered structures of the albumin secondary structure under the experimental conditions are collected in Table 3. The obtained data have shown that the saturation of BSA with the nonionic surfactants does not contribute to noticeable changes in the secondary structure of the investigated protein. On the other hand, the decrease in the α-helix content is seen for the ionic surfactants. This phenomenon is enhanced for the cationic surfactants with the increase in the pH of a solution. Thus, it can be expected that the presence of the ionic surfactants in the system can affect the biological activity of BSA.

## 3. Materials and Methods

Bovine serum albumin (BSA, lyophilized powder, ≥96%), hexadecyltrimethylammonium bromide (CTAB, BioXtra, ≥99%), sodium dodecyl sulfate (SDS, BioUltra, for molecular biology, ≥99%), and sodium cacodylate trihydrate (CACO, ≥98%) were obtained from Merck (Warsaw, Poland) and employed as received without further purification. Hexadecylpyridinium chloride monohydrate (CPC, ≥96%), polyethylene glycol sorbitan monolaurate (TWEEN 20), polyethylene glycol sorbitan monopalmitate (TWEEN 40), and polyethylene glycol sorbitan monooleate (TWEEN 80) were kindly provided by Cerko (Gdynia, Poland). Double-distilled water with conductivity not exceeding 0.18 μS cm^−1^ was used for preparations of buffer solutions. The stock solutions of the protein and all surfactants were prepared in 10 mM CACO buffer of pH 5.0 and 7.0 (all subsequent dilutions were made with this buffer). The concentration of the bovine serum albumin was measured using an extinction coefficient equal to $\varepsilon_{280}^{\mathrm{BSA}}$ = 41,180 M^−1^ cm^−1^ calculated based on the content of tryptophan ($\varepsilon_{280}^{W}$ = 5690 M^−1^ cm^−1^), tyrosine ($\varepsilon_{280}^{Y}$ = 1280 M^−1^ cm^−1^), and cysteine ($\varepsilon_{280}^{C}$ = 120 M^−1^ cm^−1^) [93,94]. A maximum absorbance value of approximately 0.08 at 280 nm (corresponding to a protein concentration of 2 µM) was used to avoid the inner filter effect. 

Steady-state fluorescence emission spectra were registered, and further emission measurements were carried out on a Cary Eclipse Varian (Agilent, Santa Clara, CA, USA) spectrofluorometer equipped with a temperature controller and a 1.0 cm multicell holder. The absorption spectra were recorded on a Lambda 650 (Perkin Elmer, Waltham, MA, USA) UV/Vis spectrophotometer. In the performed fluorescence and spectophotometric titration experiments, 2 mL of BSA at a fixed concentration equal to 2 µM was titrated with fifteen 2 μL aliquots of each surfactant’s solution (c_SDS_ = 1 mM; c_CPC_ = 4 mM; c_CTAB_ = 4 mM; c_TWEEN 20_ = 10 mM; c_TWEEN 40_ = 10 mM; c_TWEEN 80_ = 10 mM). The concentration of surfactants was varied and dependent on the observed quenching efficiency. The fluorescence intensity of the band at 348 nm—corresponding to the initial maximum emission of BSA—was used to calculate the binding constants and other parameters. The excitation wavelength was always set at 280 nm. All experiments were performed at 298 K (in the case of experiments with CPC additionally at 288 K and 308 K). All fluorescence intensity values have been corrected for the inner filter effect based on the following equation:

(4) $$F_{\mathrm{corr}}=F_{\mathrm{obs}}\cdot 10^{\frac{A_{280}+A_{348}}{2}}$$

where F_corr_ and F_obs_ correspond to corrected and observed fluorescence intensity values, respectively; while A_280_ and A_348_ correspond to absorbance values measured at the excitation and emission wavelength, respectively.

Circular dichroism (CD) spectra were recorded in 10 mM CACO buffer (pH 5.0 and 7.0) on a J-715 (JASCO Inc., Easton, MD, USA) automatic recording spectropolarimeter for pure BSA (2 µM) and all BSA–surfactant mixtures (in stoichiometric ratios equal to 1:7.5 for SDS, 1:30 for CPC and CTAB, 1:75 for TWEEN 20, TWEEN 40, and TWEEN 80) at 298 K. The spectra were recorded in the 190–260 nm wavelength range in 1 mm quartz cuvettes (the volume of sample was 0.3 mL), using a sensitivity of five millidegrees and a scan speed of 50 nm min^−1^. The effect of surfactant binding to the protein on the BSA secondary structure content was estimated from all CD spectra using the Dichroweb online server [95] with the CDSSTR analysis algorithm [96] and reference dataset 7 [97].

## 4. Conclusions

In the current work, fluorescence quenching experiments supported by UV spectrophotometry have been applied to unravel and compare the nature of binding of selected cationic, anionic, and nonionic surfactants with different head groups and chains to the circulatory protein BSA (in the cacodylate buffer; at various temperatures and pHs). Furthermore, the changes in the secondary structure of BSA on account of surfactants binding were assessed based on far-UV CD spectra. The quenching mechanism of the albumin induced by SDS, CPC, CTAB, TWEEN 20, TWEEN 40, and TWEEN 80 has been attributed to a static quenching process. The Stern–Volmer quenching constants, bimolecular quenching rate constants, binding constants, and corresponding thermodynamic parameters Δ*H*, Δ*G,* and Δ*S* were calculated, compared, and discussed. It was found that all studied surfactants interact more or less with BSA (nonionic ones exhibit lower affinity for the protein) and hydrophobic forces play a major role during the binding interaction. This phenomenon stays in line with the CD results. The interaction of cationic surfactants with BSA is a pH-dependent. The strength of the interactions increases with the increase in the pH of a solution. This points to the fact that charge–charge electrostatic interactions established between more negatively charged functional groups of amino acids at pH 7.0 (than at pH 5.0) and positively charged CPC and CTAB surfactants play a pivotal role in the stabilization of the resulting complexes. It has been proven that the presence in the system of the low molecular ligands such as SDS, CPC, CTAB, TWEEN 20, TWEEN 40, and TWEEN 80 can affect the BSA structure and thus its biological activity. On the one hand, saturation of the binding sites of BSA with surfactants hinders the binding of other ligands that reveal the affinity to the same binding site of the protein. This finding is worth taking into account when planning the use of surfactants as solubility modifiers of biologically active compounds for analyzing their interactions with the albumins. On the other hand, surfactants can selectively block the binding sites of BSA (the important component of many cell media cultures) and thus prevent binding the tested compounds with the albumin during the cytotoxicity assays. As a consequence, this can affect the concentration of free, active species.

## Figures and Tables

**Table 1:** Stern–Volmer quenching constants (*K*_SV_), linear correlation coefficients (R^2^), and bimolecular quenching rate constants (*k*_q_) recovered for the steady-state fluorescence quenching of BSA by various surfactants in 10 mM CACO buffer of pH 5.0 and 7.0 at 298 K (for CPC additionally at 288 and 308 K).

Surfactant	pH		*K*_SV_ [M^−1^]	R^2^	*k*_q_ [M^−1^ s^−1^]
CPC	5.0	288 K	0.94 × 10^4^	1.000	1.64 × 10^12^
		298 K	1.28 × 10^4^	0.998	2.25 × 10^12^
		308 K	1.98 × 10^4^	0.998	3.47 × 10^12^
	7.0	288 K	3.45 × 10^4^	0.996	5.75 × 10^12^
		298 K	5.23 × 10^4^	0.992	8.72 × 10^12^
		308 K	6.14 × 10^4^	1.000	10.2 × 10^12^
CTAB	5.0		0.81 × 10^4^	0.997	1.41 × 10^12^
	7.0		2.95 × 10^4^	0.996	4.92 × 10^12^
SDS	5.0		7.97 × 10^4^	0.989	14.0 × 10^12^
	7.0		7.65 × 10^4^	0.992	12.8 × 10^12^
TWEEN 20	5.0		0.31 × 10^4^	0.978	0.54 × 10^12^
	7.0		0.53 × 10^4^	0.987	0.89 × 10^12^
TWEEN 40	5.0		0.27 × 10^4^	0.938	0.47 × 10^12^
	7.0		0.61 × 10^4^	0.988	1.01 × 10^12^
TWEEN 80	5.0		0.34 × 10^4^	0.987	0.60 × 10^12^
	7.0		0.73 × 10^4^	0.997	1.21 × 10^12^

**Table 2:** Apparent binding constants (*K*_a_), linear correlation coefficients (R^2^) and numbers of binding sites (*n*) recovered for the steady-state fluorescence quenching of BSA by various surfactants in 10 mM CACO buffer of pH 5.0 and 7.0 at 298 K (for CPC additionally at 288 and 308 K).

Surfactant	pH		*K*_a_ [M^−1^]	R^2^	*n*
CPC	5.0	288 K	0.73 × 10^4^	1.000	0.98
		298 K	0.37 × 10^4^	0.999	0.89
		308 K	0.74 × 10^4^	1.000	0.91
	7.0	288 K	23.8 × 10^4^	1.000	1.18
		298 K	98.2 × 10^4^	0.990	1.27
		308 K	4.22 × 10^4^	0.999	0.97
CTAB	5.0		0.40 × 10^4^	0.998	0.93
	7.0		9.12 × 10^4^	0.998	1.11
SDS	5.0		77.6 × 10^4^	0.992	1.20
	7.0		85.1 × 10^4^	0.998	1.20
TWEEN 20	5.0		0.02 × 10^4^	0.993	0.70
	7.0		0.07 × 10^4^	0.998	0.78
TWEEN 40	5.0		0.04 × 10^4^	0.989	0.55
	7.0		0.07 × 10^4^	0.999	0.78
TWEEN 80	5.0		0.03 × 10^4^	0.992	0.74
	7.0		0.19 × 10^4^	0.994	0.87

**Table 3:** The secondary structure content (in %) of BSA in 10 mM CACO buffer of pH 5.0 and 7.0 at 298 K revealed from CD measurements.

System	pH	α-Helix [%]	Strand [%]	Turns [%]	Unordered [%]
BSA	5.0	61	7	12	19
	7.0	63	15	8	14
BSA + SDS	5.0	56	12	13	18
	7.0	59	15	10	17
BSA + CPC	5.0	58	9	12	20
	7.0	54	20	11	14
BSA + CTAB	5.0	60	9	10	21
	7.0	55	18	10	16
BSA + TWEEN 20	5.0	61	9	10	20
	7.0	60	15	10	16
BSA + TWEEN 40	5.0	62	10	11	18
	7.0	60	16	6	18
BSA + TWEEN 80	5.0	61	14	10	14
	7.0	54	20	11	14

## Data Availability

The data presented in this study are available on reasonable request from the corresponding author.

## Supplementary Materials

The following are available online at https://www.mdpi.com/article/10.3390/ijms23010041/s1.

## Abbreviations

BSA	Bovine serum albumin
CPC	Hexadecylpyridinium chloride
CTAB	Hexadecyltrimethylammonium bromide
SDS	Sodium dodecyl sulfate
TWEEN 20	Polyethylene glycol sorbitan monolaurate
TWEEN 40	Polyethylene glycol sorbitan monopalmitate
TWEEN 80	Polyethylene glycol sorbitan monooleate
CACO	Cacodylate buffer
CD	Circular dichroism
