# Supplementary material for: On the Effect of pH, Temperature, and Surfactant Structure on Bovine Serum Albumin–Cationic/Anionic/Nonionic Surfactants Interactions in Cacodylate Buffer–Fluorescence Quenching Studies Supported by UV Spectrophotometry and CD Spectroscopy
Source: Int J Mol Sci. 2021 Dec 21;23(1):41. doi: 10.3390/ijms23010041 (PMC8744808; doi:10.3390/ijms23010041)
Supplement: Supplementary file 1 [file ijms-23-00041-s001.zip › ijms-1489205-supplementary.pdf]

International Journal of
Molecular Sciences

MDPI

Article

On the Effect of pH, Temperature, and Surfactant Structure on Bovine Serum Albumin–Cationic/Anionic/Nonionic Surfactants Interactions in Cacodylate Buffer–Fluorescence Quenching Studies Supported by UV Spectrophotometry and CD Spectroscopy

Krzysztof Żamojć *, Dariusz Wyrzykowski and Lech Chmurzyński

Electronic Supplementary Information

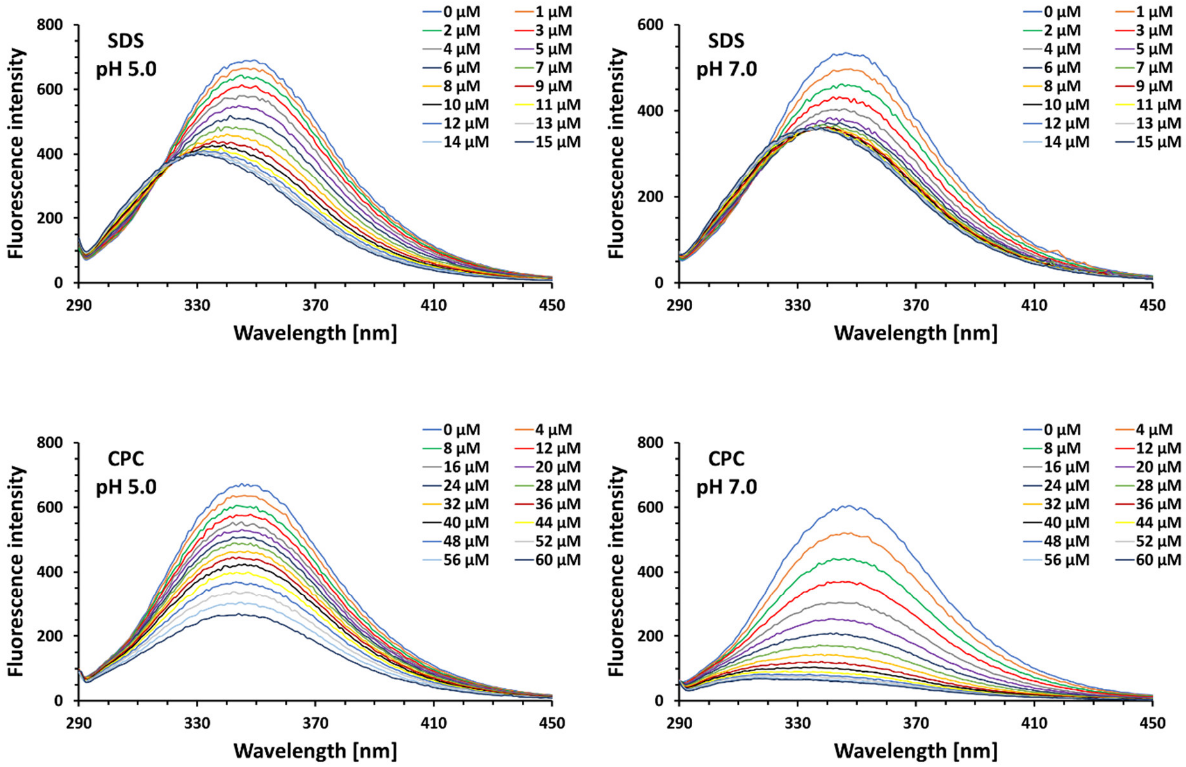

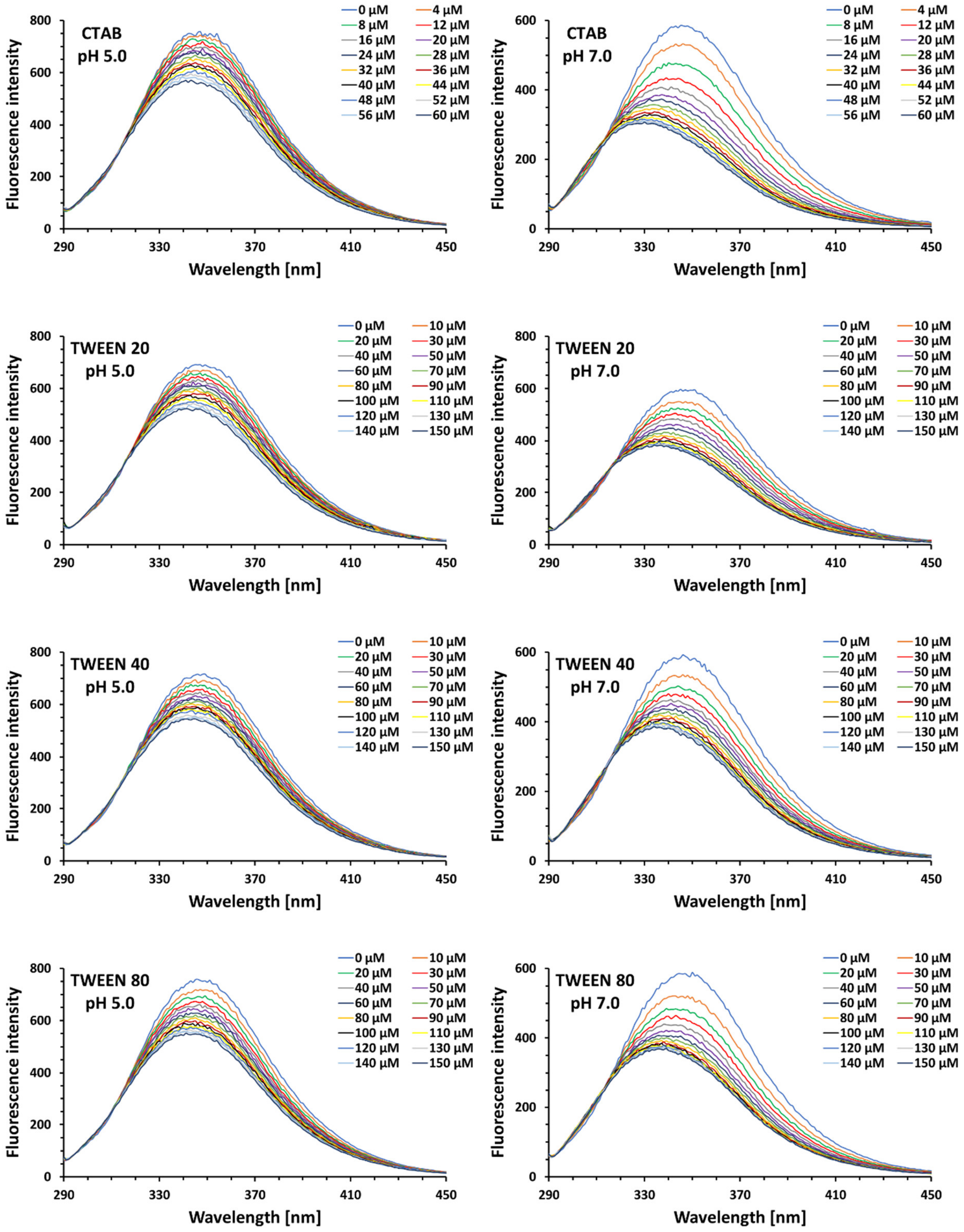

Figure S1. Fluorescence emission spectra of BSA (2 µM) in the presence of increasing concentrations of the studied surfactants in 10 mM CACO buffer of pH 5.0 and 7.0 at 298 K; λ_{ex} = 280 nm.

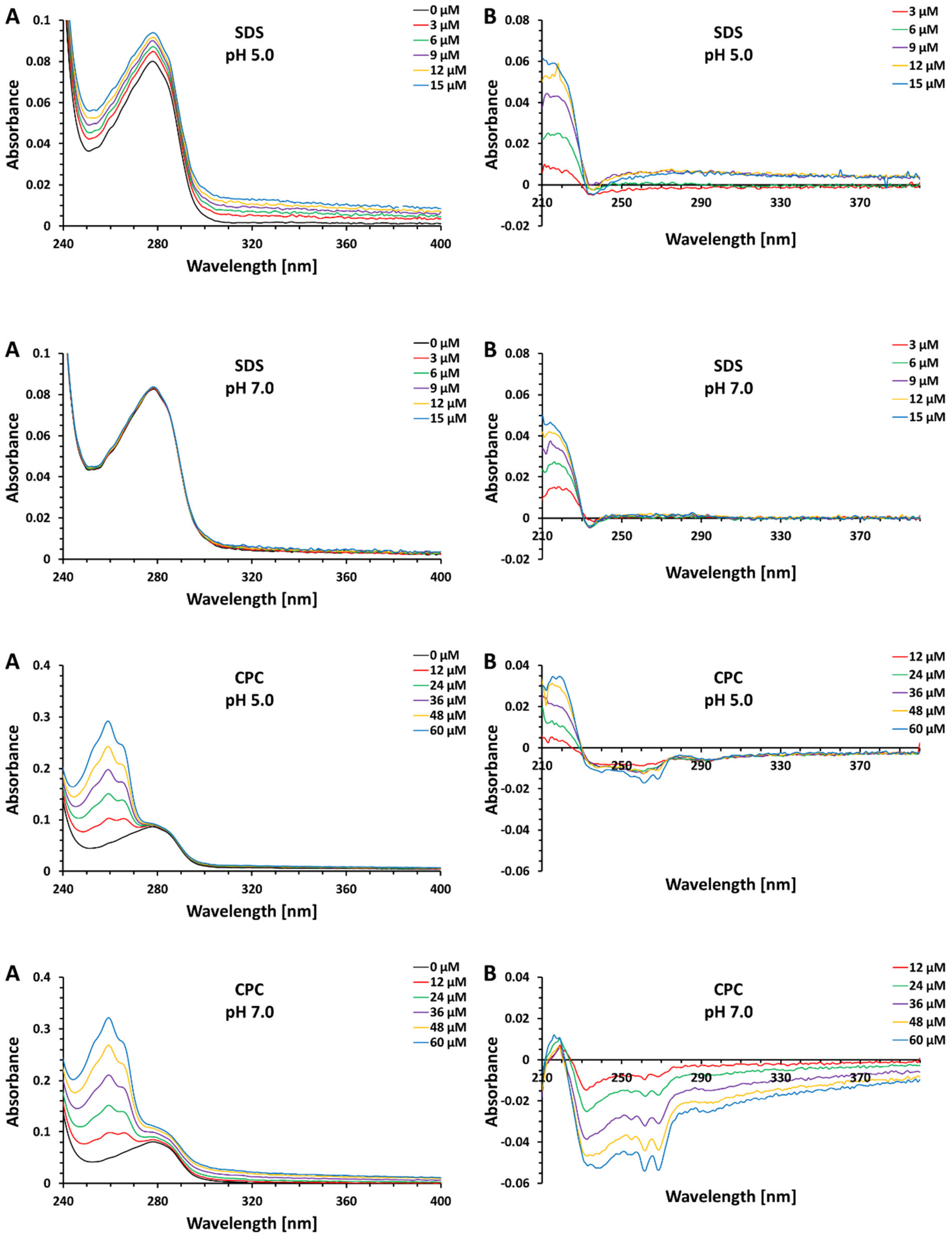
A
SDS
pH 5.0
0 µM
3 µM
6 µM
9 µM
12 µM
15 µM
Absorbance
Wavelength [nm]
B
SDS
pH 5.0
3 µM
6 µM
9 µM
12 µM
15 µM
Absorbance
Wavelength [nm]
A
SDS
pH 7.0
0 µM
3 µM
6 µM
9 µM
12 µM
15 µM
Absorbance
Wavelength [nm]
B
SDS
pH 7.0
3 µM
6 µM
9 µM
12 µM
15 µM
Absorbance
Wavelength [nm]
A
CPC
pH 5.0
0 µM
12 µM
24 µM
36 µM
48 µM
60 µM
Absorbance
Wavelength [nm]
B
CPC
pH 5.0
12 µM
24 µM
36 µM
48 µM
60 µM
Absorbance
Wavelength [nm]
A
CPC
pH 7.0
0 µM
12 µM
24 µM
36 µM
48 µM
60 µM
Absorbance
Wavelength [nm]
B
CPC
pH 7.0
12 µM
24 µM
36 µM
48 µM
60 µM
Absorbance
Wavelength [nm]

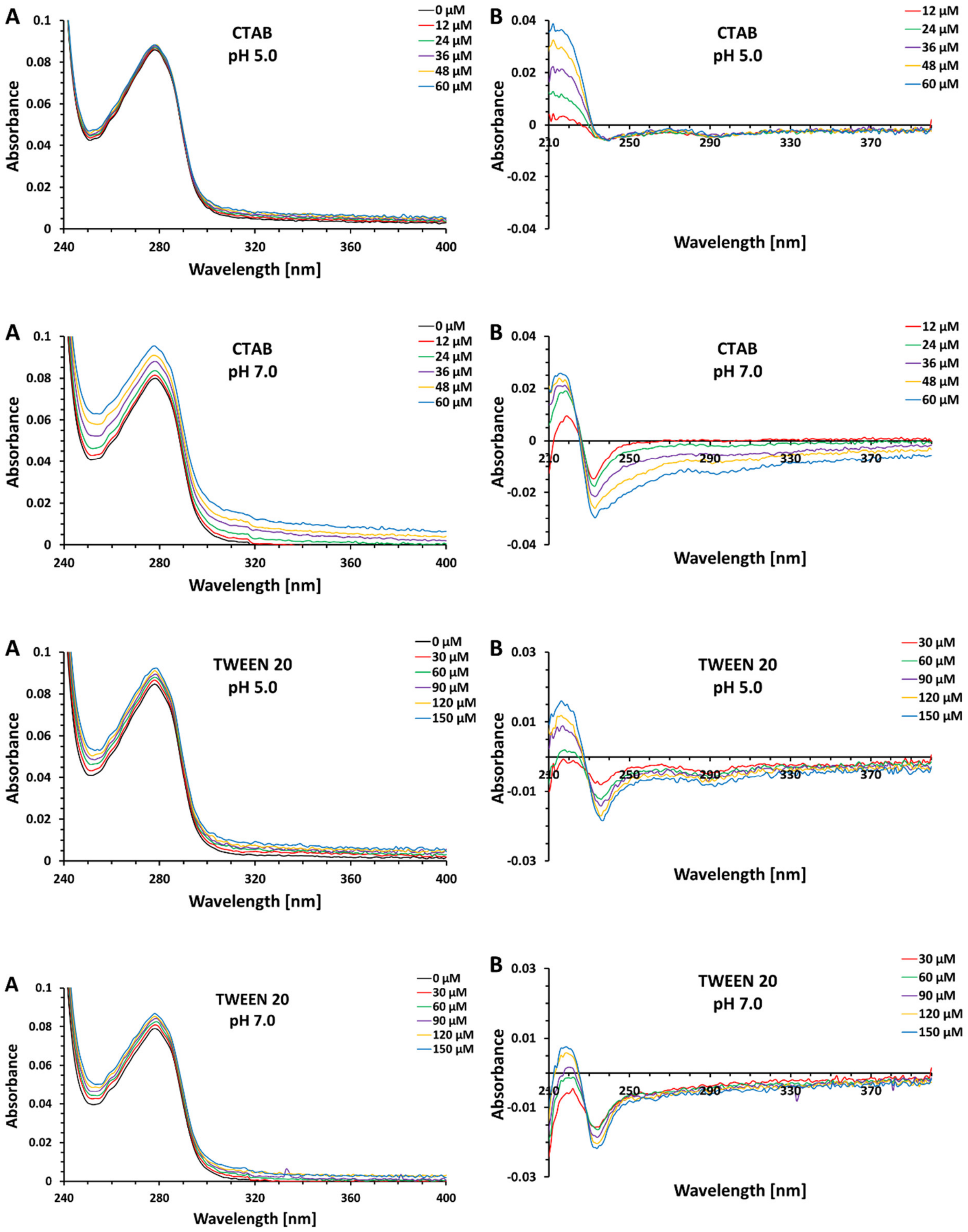
A
CTAB
pH 5.0
0 μM
12 μM
24 μM
36 μM
48 μM
60 μM
Absorbance
0
0.02
0.04
0.06
0.08
0.1
240
280
320
360
400
Wavelength [nm]
B
CTAB
pH 5.0
12 μM
24 μM
36 μM
48 μM
60 μM
Absorbance
0.04
0.02
0
-0.02
-0.04
210
250
290
330
370
Wavelength [nm]
A
CTAB
pH 7.0
0 μM
12 μM
24 μM
36 μM
48 μM
60 μM
Absorbance
Wavelength [nm]
B
CTAB
pH 7.0
12 μM
24 μM
36 μM
48 μM
60 μM
Absorbance
Wavelength [nm]
A
TWEEN 20
pH 5.0
0 μM
30 μM
60 μM
90 μM
120 μM
150 μM
Absorbance
Wavelength [nm]
B
TWEEN 20
pH 5.0
30 μM
60 μM
90 μM
120 μM
150 μM
0.03
0.01
-0.01
-0.03
Absorbance
Wavelength [nm]
A
TWEEN 20
pH 7.0
0 μM
30 μM
60 μM
90 μM
120 μM
150 μM
Absorbance
Wavelength [nm]
B
TWEEN 20
pH 7.0
30 μM
60 μM
90 μM
120 μM
150 μM
Absorbance
Wavelength [nm]

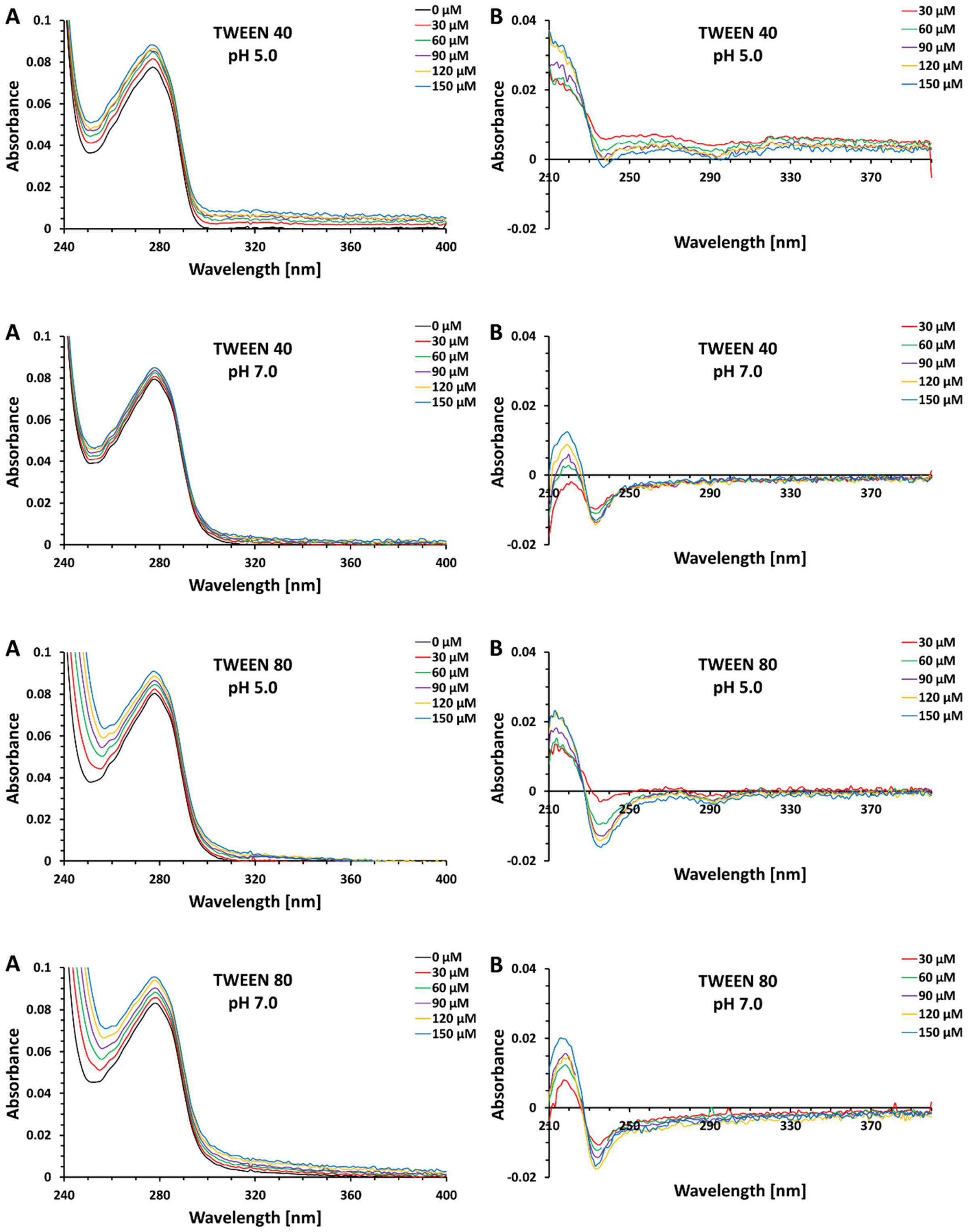

Figure S2. UV absorption spectra (A) and difference UV absorption spectra (B) of BSA (2 µM) in the presence of increasing concentrations of the studied surfactants in 10 mM CACO buffer of pH 5.0 and 7.0 at 298 K.

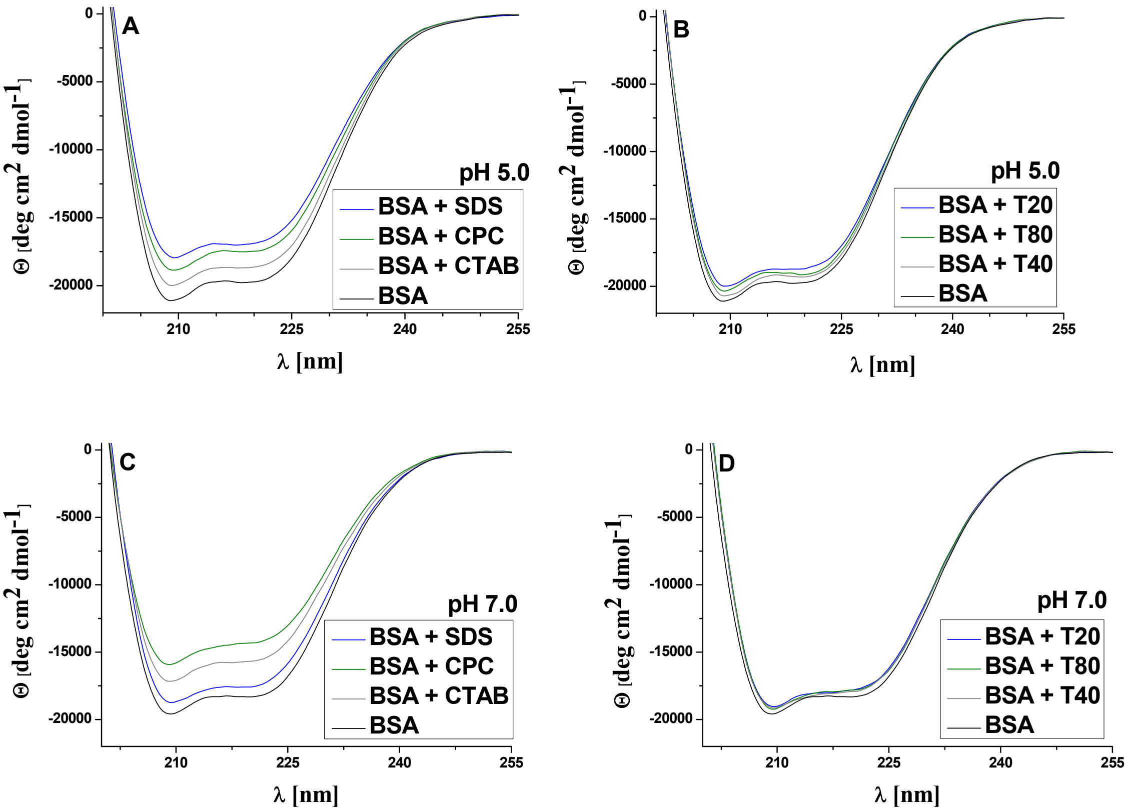

Figure S3. The CD spectra of free BSA and the solutions of BSA with the investigated surfactants mixed in 1:7.5 (for SDS), 1:30 (for CPC and CTAB), and 1:75 (for TWEEN 20, TWEEN 40 and TWEEN 80) molar ratios in 10 mM CACO buffer of pH 5.0 (A, B) and 7.0 (C, D) at 298 K.
